# Osteoarthritis and the Mediterranean Diet: A Systematic Review

**DOI:** 10.3390/nu10081030

**Published:** 2018-08-07

**Authors:** Isabel Morales-Ivorra, Montserrat Romera-Baures, Blanca Roman-Viñas, Lluis Serra-Majem

**Affiliations:** 1 Department of Rheumatology, Bellvitge University Hospital-IDIBELL, L’Hospitalet de Llobregat, 08907 Barcelona, Spain; m.romera@bellvitgehospital.cat; 2 School of Health and Sport Sciences (EUSES), University of Girona, 17190 Salt, Spain; dietmed@fin.pcb.ub.es; 3 Blanquerna Faculty of Psychology, Education Sciences and Sport (FPCEE), Universitat Ramon Llull, 08022 Barcelona, Spain; 4 CIBER Fisiopatología de la Obesidad y Nutrición (CIBERobn), Instituto de Salud Carlos III (ISCIII), 28029 Madrid, Spain; lluis.serra@ulpgc.es; 5 Research Institute of Biomedical and Health Sciences IUIBS, University of Las Palmas de Gran Canaria, 35001 Las Palmas de Gran Canaria, Spain; 6 Nutrition Research Foundation, University of Barcelona Science Park, 08028 Barcelona, Spain

**Keywords:** Mediterranean diet, osteoarthritis, systematic review

## Abstract

Osteoarthritis (OA) affects 240 million people globally. Few studies have examined the links between osteoarthritis and the Mediterranean diet (MD). The aim of this paper was to systematically review and analyze the epidemiological evidence in humans on the MD and its association with OA. A systematic search of EMBASE identified three studies that explored the association between MD and OA. Two of them were cross-sectional and the third one was a 16-week randomized clinical trial. Prisma declaration was followed to carry out this review. These studies described a positive association between a higher adherence to a MD and the quality of life of participants suffering OA. The prevalence of OA was lower in participants with a higher adherence to a Mediterranean diet. Biomarkers of inflammation and cartilage degradation related to OA were also analyzed and significant differences were detected only for IL1-α, which decreased in the MD group. Exploring the relationship between MD and OA is complex, moreover, the limited evidence and methodological differences in such studies makes it difficult to compare results. In conclusion, the three studies included in this systematic review demonstrated some relation between osteoarthritis and a Mediterranean diet. However, prospective and longer interventions are required to evaluate the long-term efficacy of the Mediterranean diet to improve symptomatology and preventing osteoarthritis.

## 1. Introduction

Dietary patterns in Mediterranean countries during the early 1960s have been linked to increased longevity and reduced morbidity as compared with those in Northern Europe and the USA [1]. More recently, several publications have demonstrated the benefits of a Mediterranean diet (MD) in reducing cardiovascular risks, certain subtypes of cancer, chronic diseases and in improving cognitive health [2,3,4,5,6]. Although definitions of MD vary, all of them include high consumption of fruit, vegetables, legumes, nuts, seeds and cereals; greater intakes of fish and seafood; moderate consumption of dairy products, poultry and eggs; as well as frequent, but moderate, intake of red wine and olive oil as the main source of dietary lipids [7,8,9,10].

Osteoarthritis (OA) affects 240 million people globally, about 10% of men and 18% of women [11]. Clinical manifestations of OA are pain, transient morning stiffness and crepitus with joint motion, all of which deteriorate daily quality of life, leading to increased morbidity and mortality [12]. The most common risk factors for OA include age, gender, prior joint injury, obesity, genetic predisposition and mechanical factors [13,14]. The link between obesity and OA is multifactorial. Historically, excessive body weight leads to wear and tear of the joint [15]. The association between overweight and hand osteoarthritis suggests that factors other than mechanical forces may play also a role [16]. Nowadays, it is accepted that obesity induces low-grade systemic inflammation caused by the secretion of proinflammatory adipokines and cytokines. These dysregulated secretions are known to contribute to joint degeneration during OA [17,18]. Moreover, it has been demonstrated that a high consumption of n-6 fatty acids results in an elevated risk of subchondral bone deterioration and that a high fat diet increases leptin levels in local cartilage tissue contributing to accelerated progression of OA [19,20].

Nowadays, the management of OA focuses on the alleviation of symptoms. International recommendations for the management of OA include three main categories: non-pharmacological, pharmacological and surgical [21]. It is widely accepted that weight loss and physical activity form part of the non-pharmacological treatment strategies for OA [22,23]. Moreover, some studies have demonstrated that nutrition can have a beneficial role in osteoarthritis [24,25,26]. Different groups have demonstrated a protector effect of the Mediterranean diet in OA because of its anti-inflammatory properties, reversion of the metabolic syndrome (MetS) and obesity and antioxidant capacity [27,28,29]. MD is rich in polyphenols which prevent inflammation and cartilage destruction, resulting in a prevention of osteoarthritis-related musculoskeletal inflammation [30]. MD also produces a lower n-6 to n-3 fatty acid (FA) ratio. Compounds derived from n-3 FA decrease gene expression of proteinases cartilage lesions and inflammatory cytokines. By contrast, a high intake of n-6 FA is suggested to induce inflammatory processes, resulting in increasing cartilage damage [31]. Some studies have reported that the intake of dietary antioxidants such as vitamin C prevents the progression of OA and drops off the prevalence of radiographic knee OA [32,33].

Hence, the aim of the current study was to systematically review and analyze epidemiological studies on the links between MD and osteoarthritis.

## 2. Materials and Methods

An EMBASE search was conducted up to December 2017 in order to identify epidemiological studies on MD and OA. The search strategy applied was as follows: (‘osteoarthritis’/exp/mj OR ‘osteoarthritis’/major focus term (mj)) AND (‘Mediterranean diet’/exp/mj OR ‘Mediterranean diet’/mj). All human epidemiological studies with full texts were considered. Additional papers were identified during the manual search process. Exclusion criteria were applied to selected studies: studies in languages other than English and studies evaluating nutrients or foods individually. The Prisma guidelines were followed during our review [34].

The quality of the studies included in the review was analyzed using the relevant tool for cross-sectional studies [35]. For the randomized controlled trial, we assessed the risk of bias (low, high or unclear) for the following domains: random sequence generation, allocation concealment, blinding of assessments, incomplete outcome data, and selective Newcastle-Ottawa Scale outcome reporting [36]. The study was classified as having a high risk of bias if any one domain was at high risk of bias and a low risk of bias if four or five domains were classified as at low risk. Otherwise, the risk of bias for the trial was considered to be unclear.

## 3. Results

**Figure 1:**
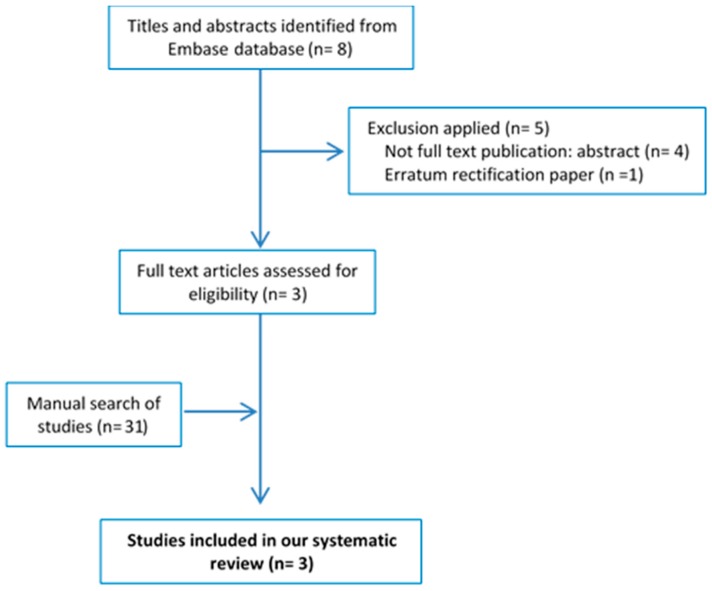
Flowchart for the systematic review.

The EMBASE search strategy (‘osteoarthritis’/exp/mj OR ‘osteoarthritis’/mj) AND (‘Mediterranean diet’/exp/mj OR ‘Mediterranean diet’/mj) yielded a total of eight articles. After applying the selection criteria, three publications were selected. The flowchart showing the procedures for selecting the studies is shown in Figure 1. Table 1 shows the main characteristics of the selected articles and Table S2 shows a summary of their main findings [37,38,39]. Two articles reported cross-sectional data from the same study, namely the Osteoarthritis Initiative conducted in the USA [38,39], while the third was a randomized controlled trial [37]. The risk of bias was fair in both cross-sectional studies and unclear in the randomized controlled trial.

.

### 3.1. Characteristics of the Study Sample

The studies were conducted between 2004–2013 in the UK and North America. The cross-sectional study was evaluated between more than 4000 participants, while the randomized control trial included 124 patients. In each study, a different principal variable of OA was analyzed: biomarkers and range of motion of knee and hip, OA prevalence and quality of life. All studies were published in last two years [37,38,39].

### 3.2. Evaluation of MD

In the Dyer et al. (2017) article, the participants in the diet group were provided with nutritional information and dietary advice regarded as adequately informative for patients from non-Mediterranean countries. All participants were asked to complete a 7-day food diary and a score to evaluate their compliance with the diet was calculated [37].

Veronese et al. (2016) analyzed the dietary patterns using the Block Brief 200 food-frequency questionnaire. Adherence to the Mediterranean diet was calculated using the scoring system proposed by Panangiotakos et al. (aMED) [38,39,40].

### 3.3. Prevalence of OA in Participants with High Adherence to MD

The prevalence of knee OA was significantly lower in participants with higher aMED (OR = 0.83; 95% CI: 0.69–0.99, *p* = 0.04). When the effects of individual components of MD and their association with the presence of knee OA were analyzed, only a higher consumption of cereals was associated with a significantly reduced probability of knee OA (OR: 0.76; 95% CI: 0.60–0.98; *p* = 0.03) [38].

### 3.4. Quality of Life and MD Adherence

Quality of life was investigated as a primary outcome through the 12-Item Short-Form Health Outcome Survey (SF-12), which has a physical (PCS) and mental (MCS) composite scale. The scores range from 0 to 100, with higher scores indicating a better quality of life. Participants with higher aMED had significantly higher SF-12: SF-12-PCS (Q5 50 ± 8.5 vs. Q1 47.2 ± 9.8 *p* < 0.0001) and SF-12MCS (Q5 54.5 ± 7.6 vs. Q1 53.2 ± 8.8 *p* < 0.0001) [38].

The presence of any depressive symptoms was analyzed using the 20-item Center for Epidemiologic Studies Depression Scale (CES-D) instrument [41]. Higher scores indicate more depressive symptoms. Participants with higher aMED had lower CES-D scores (β −0.05; 95% CI: −0.09, −0.01; *p* < 0.05) [38].

Western Ontario and McMaster Universities Arthritis Index (WOMAC) is a validated scale for assessing the presence of pain, stiffness and disability caused by OA. Participants with higher aMED had a significantly lower WOMAC in both knees for subscales of pain (Right knee −0.02 (−0.04, −0.01); *p* = 0.008)/(Left knee −0.02 (−0.04, −0.003); *p* = 0.02) and disability (Right knee −0.08 (−0.14, −0.03); *p* = 0.004)/Left Knee −0.07 (−0.14, −0.01); *p* = 0.02) but not for stiffness (*p* > 0.05) [38].

The Arthritis Impact Measurement Scale (AIMS2) is a disease-specific measure of the physical, social, and emotional well-being designed as a measure of outcome in arthritis. It encompasses nine scales: mobility, physical activity (walking, bending, lifting), dexterity, household activity, social activities, daily living activities, pain, depression, and anxiety. Dyer et al. (2017) compared AIMS2 in the diet and control groups and no significant associations emerged [37].

Dyer et al. (2017) compared the range of motion of the lower extremities in both the diet (MD diet) and control groups. Knee flexion and hip rotation in the diet group were better than those observed in the control group (122 ± 18 vs. 116 ± 29; *p* = 0.072) 52 ± 19 vs. 46 ± 24; *p* = 0.010). No significant differences between groups were observed in terms of index finger movement and hip flexion (*p* > 0.05) [37].

### 3.5. Markers of Inflammation and Cartilage Degradation

The serum cartilage oligomeric matrix protein (sCOMP) and inflammatory cytokines, chemokines and growth factors have been linked to OA. Dyer et al. (2017) analyzed different biomarkers in participants with OA in both the Diet and Control groups: sCOMP, IL-1β, IL-1α, IL-2, IL-4, IL-6, IL-8, IL-10, IFN-γ, TNF-α, VEGF, EGF and soluble receptors (IL-6sR, IL-2sR, TNF-sR1 and TNF-sR2, plus MCP-1, and MMP-9). Significant differences between groups were only detected in IL1-α (*p* = 0.019), which decreased in the diet group, but which showed no change in the control group [37].

## 4. Discussion

The findings of this systematic review indicate positive associations between MD and improved quality of life in participants with OA [37,38,39]. The prevalence of osteoarthritis was lower in participants with high aMED [38]. Biomarkers of inflammation and cartilage degradation related to OA were also analyzed and significant differences were detected for IL1-α (*p* = 0.019), which was lower in the diet group [37].

There are several physiological explanations that could explain why key components of MD might protect against OA. OA is often referred to as a degenerative joint disease. This is inaccurate since OA is not simply a process of wear and tear. Chronic low-grade inflammation has been described as a key mediator of the pathogenesis of OA [42]. In fact, elevated serum levels of the C-reactive protein, a marker of inflammation, are predictive of the development and progression of OA [43]. Inflammation in OA joints is distinct from that in rheumatoid arthritis as it is chronic, low grade and mediated by innate immunity [42]. Different studies have shown that the presence of synovial inflammation, or synovitis, in OA is associated with increased severity of joint symptoms, increased cartilage loss, decreased mobility, and elevated radiographic grades [44,45,46]. Most likely, not only local but also systemic inflammation is related to OA development [47]. Obesity induces low-grade systemic inflammation caused by the secretion of proinflammatory adipokines and cytokines, which both contribute to joint degeneration during OA [17,18]. The cytokines from the synovial fluid contribute to cartilage matrix loss by stimulating chondrocyte catabolic activity and inhibiting anabolic activity [48,49]. Thus, the known association between a higher adherence to a MD and lower level markers of inflammation could explain some of the benefits [50,51,52]. Some studies have found that consuming MD reduces weight and IL-6 levels. This effect is greater if it is accompanied by exercise [53,54].

Several studies have described an increased risk of OA in association with such metabolic risk factors as dyslipidemia, hypertension, and insulin resistance, which characterize the MetS [20,55,56,57]. The prevalence of MetS in patients with OA is 59% and in the general population 23% [55]. Patients with OA and MetS suffer an increased incidence of inflammation and pain compared to OA patients without MetS [58,59]. The treatment of MetS has been proposed as measure to delay the progression of OA [60].

The MD has been shown not only to reverse MetS [61,62,63,64], but also to improve quality of life [65,66]. On the other hand, olive oil, which is an important component of MD, has proven to be efficient in reducing pain, WOMAC scores and in improving the Health Assessment Questionnaire-Disability Index in patients with OA [67,68,69,70].

This body of evidence suggests that MD may reduce the prevalence of OA and improve the quality of life in these patients because of its anti-inflammatory effects. The absence of differences between the Diet and Control groups in terms of the biomarkers is probably a consequence of the short period of exposition to MD (only 16 weeks) or to the difficulties in adopting a MD in non-Mediterranean countries [71].

## 5. Conclusions

The findings of this systematic review should be weighed against certain limitations. Only three papers met the inclusion criteria. Two of the studies were of cross-sectional design (and evaluated the same population group) while the third was a randomized trial. There are important methodological differences in the studies that make it difficult to compare their results. In conclusion, the three studies included in this systematic review demonstrate some relation between OA and adherence to the MD. However, the epidemiological evidence is limited, and longer interventions are required to evaluate the long-term efficacy of the MD for improving symptomatology and preventing OA.

## Figures and Tables

**Table 1:** Epidemiological studies on mediterranean diet and osteoarthritis.

Author, Year	Country Population, N (sex) Age	Sample Characteristics	MD Definition. Diets and Other Interventions	Osteoarthritis	Follow-Up	Results
Dyer J et al., 2017 [37].	UK N = 99 (83% female) Age: 31–90 years.	Volunteers with a clinician diagnosis of OA. 16-week dietary intervention (N = 50) and control (N = 49).	Fruit, vegetables, legumes, olive oil, fish and shellfish, poultry, cereals (refined and whole grain), red and processed meat, animal fat, sugary drinks, sweets and pastries. Completed a 7-day food diary (as a food frequency questionnaire).	Clinician diagnosis of OA.	Food frequency questionnaire and an Arthritis Impact Measurement Scale (AIMS2) questionnaire at baseline, 2 months and 4 months (end). Subset patients (DIET = 33, CON = 28) were asked about joint range of motion (ROM) at start and end of study and to provide blood samples (DIET = 29, CON = 25) for biomarker analysis.	AIMS2 components and most biomarkers *p* > 0.05, except IL-1α in the DIET group (~47%, *p* = 0.010). ↓ Markers of cartilage degradation in the DIET group (~8%, *p* = 0.014). ↑ Knee flexion and hip rotation ROM in the DIET group (*p* < 0.05).
Veronese N et al., 2016 [38].	USA N = 4358 (2527 females) Mean age: 61.2 ± 9.1 years.	Community-dwelling participants from the Osteoarthritis Initiatives were included	The Mediterranean diet score proposed by Panagiotakos et al. was used to evaluate aMED categorized into quartiles [40].	Knee OA was diagnosed both clinically and radiologically. The presence of pain, stiffness, and physical functioning due to OA was assessed through the WOMAC. The responses for each subscale (pain, stiffness, disability) are categorized on Likert scale (0–4).	Block Brief 2000 food frequency questionnaire (FFQ) during the baseline visit.	↑ aMED ↓ prevalence of knee OA (Q4: 25.2% vs. Q1: 33.8%; *p* < 0.0001). Highest aMED ↓in presence of knee OA (OR: 0.83; 95% CIs: 0.69–0.99, *p* < 0.04). Among individual components of MD only higher use of cereals ↓ odds of knee OA (OR: 0.76; 95% CI: 0.60e0.98; *p* < 0.03).
Veronese N et al., 2017 [39].	USA N = 4470 (2605 females) Mean age: 61.3 ± 9.2 years.	Community-dwelling participants from the Osteoarthritis. Initiatives were included.	The Mediterranean diet score proposed by Panagiotakos et al. [40] was used to evaluate aMED categorized into quintiles.		Block Brief 2000 food frequency questionnaire (FFQ) during the baseline visit. Quality of life as primary outcome (SF-12) physical composite scale (PCS) and Mental composite scale (MCS). Secondary outcomes: WOMAC (pain, stiffness, disability caused by OA). Depressive symptoms (CES-D instrument) [41].	↑ aMED ↑ SF-12 PCS (Q5 50 ± 8.5 vs. Q1 47.2 ± 9.8 *p* < 0.0001) and SF-12MCS (Q5 54.5 ± 7.6 vs. Q1 53.2 ± 8.8 *p* < 0.0001). ↑ aMED ↓ WOMAC (except for stiffness). ↑ aMED ↓ CES-D (β −0.05; 95% CI: −0.09, −0.01; *p* < 0.05).

AIMS2: Arthritis Impact Measurement Scale; aMED: Adherence to the Mediterranean diet; CES-D: Center for Epidemiologic Studies Depression Scale instrument; FFQ: food frequency questionnaire; MCS: Mental composite scale of SF-12; MD: Mediterranean Diet; OA: Osteoarthritis; PCS: Physical composite scale of SF-12; ROM: Range of motion; SF-12: 12-Item Short-Form Health Outcome Survey; WOMAC: Western Ontario and McMaster Universities Arthritis Index.

## Supplementary Materials

The following are available online at http://www.mdpi.com/2072-6643/10/8/1030/s1, Table S1: PRISMA-P 2015 Checklist, Table S2: Summary of results of epidemiological studies on the Mediterranean diet and Osteoarthritis.

## Abbreviations

AIMS2	Arthritis Impact Measurement Scale
aMED	Adherence to the Mediterranean diet
CES-D	Center for Epidemiologic Studies Depression Scale instrument
FA	Fatty acid
FFQ	food frequency questionnaire
MCS	Mental composite scale of SF-12
MD	Mediterranean Diet
MetS	metabolic syndrome
OA	Osteoarthritis
PCS	Physical composite scale of SF-12
ROM	Range of motion
sCOMP	Serum cartilage oligomeric matrix protein
SF-12	12-Item Short-Form Health Outcome Survey
WOMAC	Western Ontario and McMaster Universities Arthritis Index
